# A scoping review of spirituality and religiosity in people who have had a kidney transplant

**DOI:** 10.1002/nop2.1271

**Published:** 2022-06-07

**Authors:** Amanda L. Mckie, Fellon Gaida

**Affiliations:** ^1^ School of Nursing, Midwifery and Public Health University of Canberra Canberra Australian Capital Territory Australia; ^2^ School of Nursing Griffith University Gold Coast Queensland Australia

**Keywords:** kidney failure, kidney transplant, religiosity, spirituality

## Abstract

**Aim:**

To conduct a scoping review to discover what is known about the presence of spirituality and religiosity in people who have received a kidney transplant.

**Design:**

Using Arksey and O'Malley's five‐stage framework, a scoping review of seven key databases was performed in June 2020. The scoping review follows the PRISMA extension for scoping review process.

**Methods:**

CINAHL, MEDLINE, Embase, OvidPsychINFO, JBI, Scopus and Cochrane databases were searched to identify original research, from which seven studies were identified with only four meeting the criteria. The search strategy focused on studies that were published between January 2000‐May 2020.

**Results:**

In synthesizing the available research, two key areas of interest were identified within the included studies, (1) clinical outcomes (medical adherence, renal function and transplant‐related outcomes) and (2) well‐being outcomes (locus of control and coping).

## 1 INTRODUCTION

Spirituality and religiosity are multidimensional constructs central to the human experience. In recent decades, there has been a growing interest in addressing spirituality and religiosity in health care, with evidence indicating that personal spiritual and religious practices are key determinants of health, and give meaning to the individual experience of health and illness (McCann et al., 2020). Promotion and support of spiritual and religious practices by healthcare professionals is an important component of holistic and patient‐centred care and can influence health and health outcomes in a positive way (Al‐Ghabeesh et al., 2018; Tanyi, 2002).

Evidence suggests that spirituality and religiosity can play important roles in the experience of cancer, palliative care, mental well‐being and chronic disease (Rolley et al., 2018; Vitillo & Puchalski, 2014). The impact of spirituality and religiosity on health outcomes for people living with kidney failure and following kidney transplantation, however, is an emerging area of research. Several studies identify the role of spiritual and religious beliefs in promoting acceptance, coping, compliance and support in people with kidney failure and those with a kidney transplant (Al‐Ghabeesh et al., 2018; Burlacu et al., 2019; Valizadeh Zare et al., 2018), and others suggest that people with chronic kidney disease (CKD) have higher spiritual care needs which are often left unmet (Davison & Jhangri, 2010; Valizadeh Zare et al., 2018). Some studies indicate that individuals become more spiritual after the diagnosis of a chronic disease and post‐transplantation (Glover‐Graf et al., 2007; Rafferty et al., 2015). Valizadeh Zare et al. (2018) found that having a strong sense of spirituality and religious faith was a pivotal role in providing calmness and managing fear associated with the unknown future of living with a kidney transplant.

The terms spirituality and religiosity are often used interchangeably, although there are some notable differences. Spirituality is an inherent aspect of being human and it relates to an individual's search for purpose and meaning within an experience (Rolley et al., 2018; Tanyi, 2002). Whilst spirituality can be expressed as faith, belief and religious values, it is a broader, evolving concept deeply rooted in an experience, and the interplay of actors and factors tied to that experience (Bożek et al., 2020). The concept of “transcendence” is a key feature of spirituality and signifies the relationship between the “self” and the “outside of self” (Coyle, 2002). In the health arena, this may include seeking answers, or clarity relating to a health concern or diagnosis internally from within or externally through connection to the mystical or supernatural (Koenig, 2012; Murgia et al., 2020).

Religiosity involves following a set of beliefs, practices and rituals which are often shared by a community or group (Rafferty et al., 2015; Tanyi, 2002). Belonging to a religion does not necessarily make a person spiritual and, conversely, spiritual experience may not include religious ritual and practice (Miller & Thoresen, 2003; Tanyi, 2002). Religiosity also involves transcendence through a devotion to an external higher power or God with the view that engagement with beliefs, ritual and practices will bring a person closer to the higher power (Koenig, 2012).

The relationship between spirituality and religion and health is multifactorial. Strong religious commitment is commonly thought to improve health outcomes through behavioural change such as abstinence from smoking, alcohol and risky sexual practices, all of which are promoted in some belief systems and are implicated in poorer health outcomes (Kawachi, 2020). Others point to the positive impact of spirituality and religion on psychological well‐being in terms of sense‐making and improved coping, decreased stress and improved socioeconomic position which enables people to afford high‐quality health care and other determinants of health (Ohrnberger et al., 2017; Siegel et al., 2001). Social connection is an important determinant of health and is associated with improved mental well‐being, adoption of health‐protective behaviours and anti‐inflammatory activity (Moieni & Eisenberger, 2018; Umberson & Karas Montez, 2010). Religious congregation and spiritual communities provide valuable platforms for regular social inclusion and interaction through church service or pilgrimage and shared spiritual practice (Chen & VanderWeele, 2018; King et al., 2017).

Poor health status can also impact an individual's religiosity and spirituality. People experiencing ill‐health commonly increase spiritual and religious practice and seek solace in their belief systems (Hvidt et al., 2017). Conversely, poor health outcomes may weaken religious or spiritual commitment when people disengage from religious activity during periods of illness, or if they feel that the religion to which they identify is causing harm to their health or well‐being (Maselko et al., 2012). Specific examples may include physical injury which may impact in‐person church attendance, or an individual who identifies as lesbian, gay, bisexual, transgender, intersex, queer/questioning, asexual (LGBTIQA+) denouncing commitment to a faith system which condemns their sexual or gender diversity. Similarly, people may also abandon their faith if they feel it has not led to improvement in their health state or a particular prognosis (Tarakeshwar et al., 2006).

## 2 BACKGROUND

People with kidney failure experience significant health burden and altered quality of life compared with other chronic diseases and some forms of cancer (Almutary et al., 2016; Bonner & Douglas, 2018; Davison & Jhangri, 2010). These burdens relate to the symptoms and impact of disease on quality of life indicators (Almutary et al., 2016; Yapa et al., 2020). Kidney failure encompasses a range of disorders that affect the kidney's ability to filtrate and remove wastes from the body and is usually irreversible and progressive with disease onset (Kidney Health Australia, 2019). Individuals with later‐stage kidney failure, will typically require renal replacement therapy (RRT) via haemodialysis (HD), peritoneal dialysis (PD) or kidney transplantation, otherwise the clinical manifestation will become life threatening (Kidney Health Australia, 2019). The choice and nature of RRT are impacted by several factors including the individual's medical and surgical history, the presence of comorbidities (including cardiovascular disease), and the individual's social circumstances including employment, housing and support networks (Bonner & Douglas, 2018; Daugirdas, 2019). Individuals who receive a kidney transplant require lifelong and ongoing healthcare engagement, and need to make significant lifestyle adjustments through dietary restrictions and strict medication compliance to prevent graft rejection (Bonner & Douglas, 2018; Yang et al., 2020).

Contemporary models of health‐care are veering away from merely biomedical approaches to hybrid bio‐psycho‐social models where care is participatory, and the people and their families have greater agency in how health‐related decisions are made and how health care is delivered (Siffels et al., 2021). Renal nurses have a significant role in the care of individuals with kidney failure and those who have received a kidney transplant. It has been well established that renal nurses care for people with kidney failure over an extended period (Bonner, 2007; Hayes & Bonner, 2010). Approximately 61% of Australians and 85% of people globally are affiliated with a religion or spiritual belief system (ABS, 2016; Hackett et al., 2015). As such, the unique and ongoing therapeutic relationship between renal nurses and the people provides an opportunity for the identification of spiritual and religious needs and preferences so that they can be meaningfully incorporated into the holistic care of the people (Cousins et al., 2020; Jugjali et al., 2018). Evidence suggests that when a professional relationship has been established between the nurse and people, there is an increase in treatment adherence and compliance, both of which are essential in the optimal management of complex conditions such as kidney failure and kidney transplantation (Cousins et al., 2020; Richardson et al., 2015).

Currently, there is one systematic review of studies that explores spirituality in people with kidney failure and receiving HD (Al‐Ghabeesh et al., 2018). This study aimed to establish a connection between spirituality, health outcomes and general well‐being, although it is not clear if studies of people who received a kidney transplant were included. In another systematic review, Burlacu et al. (2019) identified a positive relationship between quality of life, spirituality and religiosity in people receiving HD. The review aim of Burlacu et al. (2019) was to synthesize the tools used to measure spirituality and religiosity and identify correlations in levels of quality of life in people receiving HD. This review did not include people who had received a kidney transplant (Burlacu et al., 2019). The current literature related to kidney transplant recipients and spirituality and religiosity is minimal, and although there is an abundance of research into how spirituality and religiosity impact health outcomes in the broader sense, there is very little research relating to kidney recipients and health outcomes specifically. Gaps in the current literature suggest that spirituality and religiosity in people with CKD who have received a kidney transplant are not well explored and understood.

### 2.1 Research question

Given the impact that kidney failure and transplantation can have on an individual and the long‐term holistic care requirements of people with kidney failure, this scoping review aimed to explore what is known about spirituality and religiosity in people who have received a kidney transplant using the available evidence. This scoping review endeavoured to address this question through a map of the current literature and the identification of evidence gaps to inform future research.

## 3 THE STUDY

### 3.1 Design

The review was conducted in line with Arksey and O'Malley's (2005) scoping review methodological framework and followed the PRISMA‐ScR checklist (Tricco et al., 2018; see Supporting information Table S1).

### 3.2 Methods

Scoping reviews are used to map a field of study to identify what is known about a topic and to identify important evidence gaps which may require further research (Arksey & O'Malley, 2005). Arksey and O'Malley's (2005) framework for scoping reviews involves, (i) identifying the research question, (ii) identifying studies that are relevant to the research question, (iii) study selection, (iv) charting the data and (v) assembling, reviewing and reporting of the results. This framework was selected as it allowed for broad concepts and a variety of different study designs to be included in the review process.

### 3.3 Inclusion criteria

This review focused on research pertaining to spirituality, religion, faith, belief systems and kidney transplantation. All participants within included studies were required to have received a kidney transplant. The search was then limited to English language studies published between January 2000–May 2020. The 20‐year period search strategy was selected given much of the literature related to the concept of spirituality and religion within chronic disease management and transplantation has been published during this time period. This review included studies that were methodologically qualitative and quantitative (see Table 1).

**TABLE 1:** Inclusion and exclusion criteria

Selection criteria	Inclusion criteria	Exclusion criteria
Language	English	Non‐English
Dates	Publications from Jan 2000‐2020	Publications before 2000
Study types	Quantitative research Qualitative research Systematic designs	Conference abstracts, reports and case studies, news articles and editorials Unpublished primary studies Studies that were ambiguous and vague about spirituality and religiosity
Topics	Spiritually, spiritual, faith, belief, values, religion, religious, renal, kidney, transplant, transplantation	Specific religious domains and religious affiliations

### 3.4 Search strategy

**FIGURE 1:**
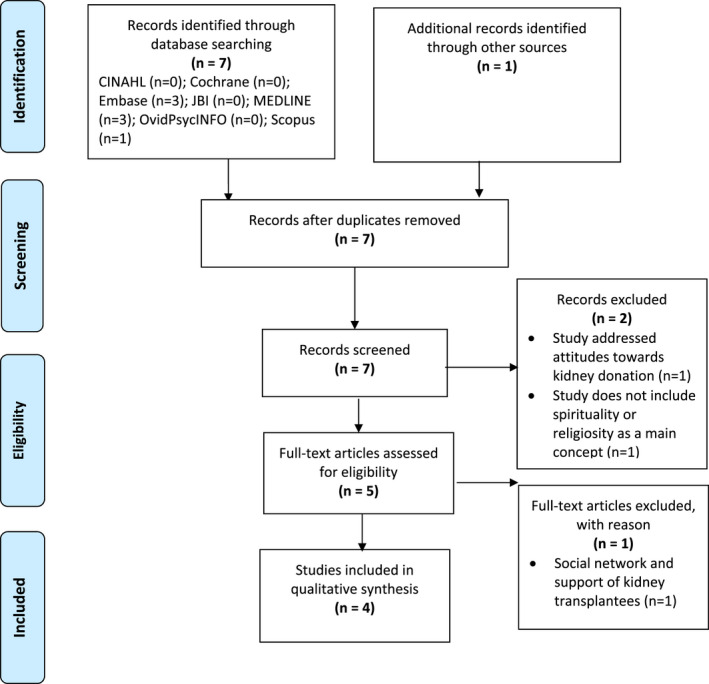
Study selection flow chart. From Tricco et al. (2018)

In June 2020, CINAHL, MEDLINE, Embase, OvidPsychINFO, Scopus, JBI and Cochrane databases were searched using a range of keywords to promote inclusiveness and sensitivity within the search (see Table 2). The reference lists of the selected studies were also inspected for other studies which would be considered appropriate to include in the review. Both authors read all identified abstracts to ensure that inclusion and exclusion criteria were met. Figure 1 highlights the search collection and screening process following the PRISMA guidelines (Tricco et al., 2018).

**TABLE 2:** Search strategy embase using keywords and mesh

Search strategy		Results
1	“spirituality” OR “spiritualistic” OR “spiritualities” OR “spiritual” OR “religion” OR “religious” OR “religiosity” OR “faith” OR “belief” OR “belief systems”	160,760
2	“renal transplant” OR “renal transplantation” OR “kidney transplant” OR “kidney transplantation”	172,685
3	1 AND 2	10
4	Limiters‐ English language	10
5	Limiter‐Date of Publication January 1, 2000 to June 1, 2020	8
6	3 AND 4 AND 5 AND Journal article or Review	3

### 3.5 Study Selection

Within the screening process, seven studies were identified and considered relevant. Of those seven studies, one was a duplicate and was removed from inclusion. One additional study was identified for inclusion when searching the reference list of the original studies. Figure 1 highlights the screening process and search strategy using the PRISMA extension for scoping review checklist (Tricco et al., 2018). Both authors independently screened the titles and abstracts of the seven screened studies assessed for eligibility. Two studies were removed as they did not address spirituality or religiosity as the main concept. Five remaining studies were included in the full text review with only four meeting the final inclusion criteria. The two authors independently reviewed all studies, and consensus was reached in all four studies.

### 3.6 Data extraction

Table 3 details the study characteristics for each of the four included studies. Characteristics include authors, year of publication, study location, aim, study design, sample size and the key findings (see Table 3).

**TABLE 3:** Summary of studies included in the review

Lead Author/Year	Country	Setting	Aim	Design	Sample	Key demographics	Outcome measures	Instrument	Major Findings	Key themes
Martin and Sachse (2002)	USA	Mid‐southern transplant clinic Urban University	To examine the spiritual perspective of women who have received a renal transplant	Descriptive correlational	28 women who have received a renal transplant	All female 64% “black” 93% high school graduates 100% had incomes of <$10,000 per year	Spiritual perspective, spiritual well‐ being, religious well‐ being and existential well‐being	Spiritual perspective scale (SPS) and Spiritual wellbeing scale (SWS)	High levels of spiritual well‐being were identified in people with a renal transplant	Well‐being (+)
Moysés Bravin et al. (2017)	Brazil	Multidisciplinary kidney transplantation service in Large Public Hospital in countryside of large city	To measure the influence of spirituality in renal function of people who have received a renal transplant.	Cross‐sectional study.	81 people who have received a renal transplant.	18 years or older, >30 days and < 60 days post‐transplant. Single organ transplant only. 53% women, mean age 42. Most had completed high school	1. Evolution of renal function (creatinine Cockcroft‐Gault formula) at baseline, 3, 6, 9 and 12 months post renal transplantation 2. Loss of allograft	Duke University Religion Index (DUREL)	People who were considered “spiritualised” displayed improved renal function and zero allograft loss. Significant results demonstrated after the ninth month	Clinical findings (+) Adherence (−) State of well‐being (−)
Saadatpanah et al. (2018)	Iran	Organ transplantation center at large hospital	To identify the relationship between coping and spiritual health in people with a renal transplant	Descriptive correlational study	169 people who have received a renal transplant	Older than 18 years of age, approximately even male to female ratio. 68% were married. 36% had a high school education	Spiritual health Coping	The spiritual health questionnaire (SHQ)	A significant relationship was identified between a person's spiritual well‐being and coping mechanism	State of well‐ being (coping)
Silva et al. (2016)	Brazil	University hospital kidney transplant center	To investigate how self‐efficacy, locus of control and religiosity is associated with adherence to immunosuppressive medications in post‐transplant recipients	Cross sectional observational study	88 people who have received a renal transplant	18 years or older, at least 12 months post‐transplantation. 73% were males. Mean age of 47.2 years. 2/3 were retired	Adherence to immunosuppressive medication	Duke University Religion Index (DUREL)	Adherence to immunosuppressive medication was associated with high levels of self‐efficacy and religiosity	Adherence state of well‐being

## 4 ANALYSIS

The authors looked for common aspects across the included studies from which common themes were generated. This resulted in the identification and naming of two common themes across the four included studies, (i) clinical outcomes and (ii) well‐being outcomes. Subcategories were then identified within each of the key themes identified: medication adherence, renal function, adverse transplant outcomes, locus of control and coping.

## 5 RESULTS

Only four studies published between January 2000‐May 2020 were included in this scoping review. Two of the studies were cross‐section research designs (Moysés Bravin et al., 2017; Silva et al., 2016), and both were conducted in Brazil. The remaining two studies were descriptive‐correlational study designs (Martin & Sachse, 2002; Saadatpanah et al., 2018) and were conducted in the USA and Iran, respectively. Spirituality and religiosity were measured across the four included studies using several instruments (see Table 3 for an overview of included studies). Moysés Bravin et al. (2017) and Silva et al. (2016) both used the Duke University Religion Index (DUREL), whereas Saadatpanah et al. (2018) used the Spiritual Health Questionnaire. Martin and Sachse (2002) used The Spiritual Perspective Scale and The Spiritual Wellbeing Scale. The results of this scoping reveal they found two key areas of interest, (i) clinical outcomes including medical adherence, renal function and adverse transplant outcomes and (ii) well‐being outcomes which included locus of control and coping.

### 5.1 Clinical outcomes (medication adherence, renal function and adverse transplant outcomes)

The acknowledgement of the important role that spirituality and religiosity play within clinical outcomes emerged from the studies. Two studies identify clinical outcomes in their methodology. Moysés Bravin et al. (2017) observed clinical outcomes in terms of medication adherence, renal function and transplant‐specific complications. Silva et al. (2016) focused on medication adherence alone. Medication adherence relates to the extent to which an individual's medication‐taking behaviour (including commencement, timing and cessation of medication) aligns with advice provided by the treating healthcare practitioners (Lehmann et al., 2014). Renal function relates to the normal functioning of the kidneys as measured through glomerular filtration rate (Daugirdas, 2019). For this review, adverse transplant outcomes include biopsy‐proven cellular rejection, loss of allograft and death.

### 5.2 Medication adherence

Increased adherence to medication and treatment regimens was reported in two of the studies. Moysés Bravin et al. (2017) measured medication adherence via serum levels of common immunosuppressive therapy (cyclosporin, tacrolimus, sirolimus and everolimus) at baseline, as well as 3, 6, 9 and 12 months post‐transplantation, with an evaluation of adherence determined using the Basel Assessment of Adherence to Immunosuppressive Medication Scale (BAASIS). The study did not observe a correlation between spirituality and adherence to immunosuppressive therapy.

Silva et al. (2016) measured medication adherence using three tools, (i) self report using the BAASIS, (ii) a collateral report provided by treating health professionals and (iii) serum levels of common immunosuppressive therapy (cyclosporin, tacrolimus and sirolimus). Results from the study indicate that medication adherence is correlated with intrinsic religiosity specifically, compared with non‐adherent participants. No correlation was observed between adherence and other measures of religiosity including organized and non‐organized religions. Organized religion includes a religion that is arranged by a formal official body and has a strict set of rules, whereas non‐organized religion is less formally governed. Silva et al. (2016) suggest intrinsic religiosity is internally rather than externally motivated which may increase internal motivation to comply with medication regimes.

### 5.3 Renal function

Renal function was observed in two of the studies (Moysés Bravin et al., 2017; Silva et al., 2016). Moysés Bravin et al. (2017) measured renal function through creatinine clearance levels at baseline and at 3, 6, 9 and 12 months post‐transplantation using the Cockcroft‐Gault formula. Participants who were in the spiritualised category demonstrated improved renal function at 12 months post‐transplantation almost double the creatinine clearance than participants in the less‐spiritualised group (61.5% vs. 34.5%). Decreased renal function in the less spiritualised group could not be explained by any other clinical factors (Moysés Bravin et al., 2017). Moysés Bravin et al. (2017) suggest that participants in the spiritualised group were more likely to adhere to treatment regimens and this directly led to improved renal function (Moysés Bravin et al., 2017). Silva et al. (2016) found religiosity was also indirectly correlated with improved renal function. As mentioned previously, participants who were identified as being intrinsically religious were more likely to be adherent with immunosuppressive medication. Similarly, participants in the adherent group had improved renal function (creatinine 1.3/0.3) compared with participants in the non‐adherent group (1.7/0.7).

### 5.4 Adverse transplant outcomes

Only one study explored the relationship between spirituality and adverse transplant outcomes. Moysés Bravin et al. (2017) measured proportions of biopsy‐proven acute cellular rejection, loss of allograft and death in each of the two study groups (spiritualised and less‐spiritualised). No people in the spiritualised group experienced loss of allograft, whereas 6.9% experienced graft loss in the less spiritualised group (*p* 0.12+). No difference in deceased (death) outcomes was observed between the groups.

### 5.5 Wellbeing outcomes

#### 5.5.1 Locus of control

Evidence suggests that an individual's perceived locus of control can impact emotional adjustment pre‐ and post‐organ transplantation as well as survival rates post‐transplantation (Burke, 2008; Burker et al., 2005). Locus of control is a psychological concept defining the degree of control an individual has over their life (Basińska & Andruszkiewicz, 2016). Individuals with an internal locus of control perceive that their health is directly attributable to their actions, whereas individuals with an external locus of control are influenced by a host of external factors including luck, fate and injustice (Burke, 2008). Spirituality is typically associated with a higher internal locus of control. Silva et al. (2016) explored the relationship between religiosity and locus of control in people who had received a kidney transplant, and discovered religiosity is commonly associated with an external locus of control. Participants who tended toward “chance” (external) locus of control often associated with extrinsic religiosity were more likely to be non‐adherent to immunosuppressive medication following kidney transplantation.

#### 5.5.2 Coping

The relationship between coping and spiritual health was identified in all four studies. Coping is defined as the emotional, intellectual and behavioural effort required to adapt to and manage change (Livneh, 2016). Moysés Bravin et al. (2017) explored coping in terms of the experience of depression and anxiety and evaluation of social supports. Although spirituality, religiosity and social support mechanisms can improve coping in times of stress, Moysés Bravin et al. (2017) found no difference in depression and anxiety scores, and social support between the spiritualised and non‐spiritualised groups.

Martin and Sachse (2002) explored the impact of spiritual perspective and spiritual well‐being in women who had received a kidney transplant. Although coping was not an outcome measured in the study, the authors did acknowledge the women as “survivors” as they had endured the burden and life‐threatening experiences of CKD and HD treatments, and were now managing the restrictions associated with transplantation. Martin and Sachse (2002) concluded that spiritual perspective and spiritual well‐being are positive psychological tools that individuals may draw on to cope with transplantation and associated life events. Additionally, efforts to reduce adverse outcomes following a kidney transplantation should include considerations of social support structures and psychological well‐being.

Silva et al. (2016) suggest the inherent desire to “take care of one's self” was much stronger in people with a kidney transplant. These participants consequently scored higher on the DUREL intrinsic religiosity scale. The concept of coping in terms of self‐efficacy and the ability of participants to overcome difficulties and stressors was identified in this study. The findings indicate that the relationship between self‐efficacy, religiosity and medication adherence in people who have received a kidney transplant is multi‐directional and complex. Silva et al. (2016) suggest that religion has an important role in coping and improved quality of life for people. In the study, it was observed that higher intrinsic religiosity increases perceptions of self‐efficacy.

Saadatpanah et al. (2018) investigated the relationship between coping and spiritual health in participants who had undergone kidney transplantation. Coping was measured as five dimensions, (i) conscious acceptance of the existing situation, (ii) the conceptualisation of self‐care commitment, (iii) conscious tolerance of difficulties, (iv) focus on supportive encouragement and (v) spiritual tolerance. There was a statistically significant relationship between spiritual health and coping (*p* < .001, *r* + 0.37, CI 95%), with the view that increased spirituality can predict better coping in people with a kidney transplant. Saadatpanah et al. (2018) also identified that marital support in combination with spiritual health was an important predictor in managing kidney transplantation as participants feel more supported and less likely to encounter risks concerning their treatment regime.

## 6 DISCUSSION

In this scoping review, we identified only four studies that explored spirituality and religiosity in people who have received a kidney transplant. Our findings identified that spirituality and religiosity have an impact on both clinical and well‐being outcomes in people who have received a kidney transplant. Although the body of research exploring the association between spirituality and religiosity in people with a kidney transplant is sparse, our findings suggest that the impact of spirituality and religiosity on clinical and well‐being outcomes should be better understood by nursing and health professionals. Spirituality and religiosity should also be considered as a part of a holistic approach to the care of people undergoing kidney transplantation.

This scoping review identified that spirituality and religiosity influence kidney transplant outcomes primarily through increased adherence to immunosuppressant medication and improved coping. The role of spirituality and religiosity in medication adherence has been well researched within other areas of health, including hypertension, HIV, mental health and diabetes management, but with varying effects (Abdul Wahab et al., 2021; Gültekin & Kavak Budak, 2022: Oji et al., 2017; Saffari et al., 2019). Several studies suggest that people with high levels of spirituality and religiosity were more likely to be non‐adherent to anti‐hypertensive medications as the belief and trust in divine healing and supreme intervention overpowered the motivation required for medication self‐management. (Kretchy et al., 2013; Wanyama et al., 2007). Similarly, others have found that some people did not feel individual responsibility to adhere to complex medication regimes as they believed God would manage their condition which, in some cases, led to significant worsening of their condition and other complications (Johnstone et al., 2008; Kretchy et al., 2013).

Although there are several studies that examine locus of control, coping and acceptance in people with kidney failure receiving HD treatment (Kohli et al., 2011; Mahmoud & AbdElaziz, 2015), this scoping review found that well‐being outcomes such as locus of control and coping are strongly linked to spirituality and religiosity of people in receipt of a kidney transplant. Findings suggest that a strong sense of faith can help people process and accept a diagnosis of kidney failure and the acceptance of HD treatment (Mahmoud & AbdElaziz, 2015). Moreover, the feeling that God is in control of a person's future can provide a great sense of comfort and security to some people (Aghakhani et al., 2014; Biniaz et al., 2018).

The role of faith in alleviating the burden of kidney failure and HD is evident in many studies (Aghakhani et al., 2014; Al‐Ghabeesh & Suleiman, 2014; Biniaz et al., 2018; Shahgholian & Yousefi, 2015). Although faith is synonymously linked with religion and spirituality, they are different concepts. Faith is a construct of having belief and trust in something, and this may or may not be spiritual or religious in nature (Harris et al., 2018). In these studies, some people identified that their faith provided comfort and support in managing the distressing symptoms associated with HD (Aghakhani et al., 2014; Al‐Ghabeesh & Suleiman, 2014; Biniaz et al., 2018; Shahgholian & Yousefi, 2015). Conversely, others felt their kidney failure diagnosis was a punishment from God for evil in their past lives, and that the burden of HD was required to correct their actions (Lin et al., 2005; Vafaei & Nobahar, 2017).

In line with our findings, others agree that there is a conceptual overlap between locus of control and spirituality/religiosity (Olagoke et al., 2021). This scoping review found that well‐being outcomes such as locus of control and coping are strongly linked to the spirituality and religiosity of people in receipt of a kidney transplant. We found that locus of control has a significant impact on adherence to immunosuppressive medications post‐kidney transplantation. There are synergies between the findings of this study and findings from Kinney et al. (2002) who found that African American women with a high God Locus of Control (external) who were at high risk for breast cancer were less likely than those with a low God Locus of Control to adhere to recommended clinical breast examination and mammography. The authors suggested that participants felt God had more influence on breast cancer morbidity than biomedical or physiological characteristics.

Whilst this scoping review focuses on religiosity and spirituality, there are other factors which also impact coping and medication adherence post‐renal transplantation which require acknowledgement but are beyond the scope of this study. Socioeconomic status and transplant‐related stressors are known to impact coping pre‐ and post‐kidney transplantation. White and Gallagher (2010) found that higher levels of employment and education were associated with higher quality‐of‐life coping scores, and suggest that employment and education can increase adjustment to transplant‐related incapacity and the associated psychological impacts. Lee and colleagues (2017), however, identified a broad range of stressors which impact coping in transplantation recipients including long wait times, deterioration in quality of life and uncertainty about the future.

Gill (2012) highlighted that the relationship between the donor and recipient had a significant impact on recipient coping post‐transplant. In this study, some living donor (LD) participants identified a positive experience given both the donor and recipient, who were often family members or spouses were “in it together”. Other recipients identified challenges with recipient feelings of guilt when their LD underwent an operation that they did not medically require. Guilt was also experienced by donors whose organ failed to function following transplantation. Lai et al. (2020) also acknowledged that guilt is also commonly experienced by deceased donor recipients when they receive a healthy organ in the context of another family's tragedy.

Age, social support and health literacy are correlated with immunosuppressant medication adherence. Although the studies included within this scoping review involved participants over the age of 18 years, the findings related to treatment adherence are broadly extrapolatable across age groups. Children and young people experience higher rates of graft rejection compared with any other age group (Boucquemont et al., 2019). Findings suggest that this is due to forgetfulness and poor organization and planning, a lack of perceived control over health outcomes and concerns about the impact of medications on appearance and social relationships (Boucquemont et al., 2019; Tucker et al., 2001 & Tielen et al., 2014). Kripalani et al. (2010) adds that older people are also at risk for medication non‐adherence and transplantation as a result of forgetfulness, cognitive decline or fatigue with taking multiple medications over time.

Prihodova et al. (2014) found that family support was correlated with excellent medication adherence. Authors suggested this is due to family members providing regular reminders to the transplant recipient to take medication and being readily available to collect medications from the pharmacy or support the recipient to access regular post‐operative health care. In terms of health literacy, Mayo‐Gamble and Mouton (2018) suggest that miscommunication between health providers and people who are unable to read, understand and seek clarification around medication instructions, are most at risk of medication non‐adherence.

In conducting this scoping review, search terms remained broad to capture any studies exploring the relationship between spirituality, religiosity and kidney transplantation. Although no particular population groups were specified within the search strategy, the translation of findings may inform care provided to populations who are particularly religious or spiritual in nature as well as specific ethnic and cultural groups who experience disproportionate rates of kidney failure and kidney transplantation.

The highest burden of kidney failure is concentrated in the three lowest quintiles of the Socio‐Demographic Index: Oceania, sub‐Saharan Africa and Latin America (Bikbov et al., 2020). Similarly, these regions also account for the highest levels of self‐reported religiosity and spirituality in the world (Bragazzi & Puente, 2013; Höllinger & Makula, 2021). Whilst availability and safety of kidney transplantation may not correlate with transplant need in some of these regions (Akoh, 2011), given the risk for developing kidney failure in these populations coupled with the value these population groups place on religion and spirituality, consideration of spirituality and religiosity in the care of many cultural groups may impact health outcomes for people diagnosed with kidney failure.

Indigenous Australian adults are twice as likely to have kidney failure than non‐Indigenous Australians, and rates of treatment for kidney failure are increasing in this population (Australian Institute Health and Welfare, 2020). Sivertsen et al. (2020) explored the relationship between spirituality and coping in Aboriginal people, and identified that an individual's spiritual well‐being includes the connection between the person, family and community. According to Terpstra et al. (2021), spirituality is perceived uniquely within Indigenous populations and is closely linked with their overall quality of life. Given that spirituality is considered a powerful determinant of health for Indigenous populations, more depth in understanding is required to meaningfully integrate spirituality in health‐care programs for Indigenous people (Harrington, 2016; Sivertsen et al., 2020).

## 7 LIMITATIONS

The strength of this scoping review lies in the structured approach to the literature regarding spirituality and religiosity. This scoping review followed Arksey and O'Malley's (2005) framework for conducting a scoping review, and the PRISMA extension for scoping review checklist (Tricco et al., 2018). The inclusion of both qualitative and quantitative studies allowed a broad and in‐depth approach to understanding the topic. This review focused on the concept of spirituality and religiosity regardless of the religion identified by the participants within the studies. Nevertheless, this review does have some limitations. This review only included studies published in English language which may have resulted in some studies being excluded. More search terms could have been used to elicit additional studies. Additionally, given this is a scoping review, we did not quality appraise any of the studies. Some of the studies included in the review are from countries with high levels of spiritual and religious engagement where the role of the family is central to the experience of health or ill‐health.

## 8 CONCLUSION

This scoping review demonstrated the impact of spirituality and religiosity on health outcomes for people who have received a kidney transplant. Only four studies which focused on spirituality and religiosity in people who have received a kidney transplant were included in this scoping review. Two common key themes emerged from the review, (i) clinical outcomes including medication adherence, renal function and adverse transplant outcomes and (ii) well‐being outcomes which include locus of control and coping. These two important themes provide insight into the importance and complexity of spirituality and religiosity in people who have received a kidney transplant. Further research is required to understand how spirituality and religiosity can be effectively integrated into standard nursing care to ensure that the individual needs of the patient are met and transplant outcomes and quality‐of‐life levels are optimized.

The findings from this scoping review have identified the role that spirituality and religiosity play in influencing clinical and well‐being outcomes for people who have received a kidney transplant. Health professionals have an important role in providing person‐centred care to people with kidney failure and following a kidney transplant. Given the impact that kidney transplantation can have on an individual and their family unit, renal nursing and health care teams should consider incorporating spirituality and religiosity into a patient's care plans as standard practice. Although the evidence base remains scant, the results from this scoping review can be used to inform future research to shape the development of post‐transplantation care packages, and provide professional education opportunities for renal health care providers.

## AUTHOR CONTRIBUTIONS

Amanda L. Mckie led the review process. Amanda L. Mckie and Fellon Gaida drafted, reviewed and approved the manuscript.

## CONFLICT OF INTEREST

No conflict of interest has been declared by the authors.

## ETHICAL APPROVAL

Ethical approval was not required for this scoping review.

## Supporting information


Table S1

## Data Availability

As a scoping review, we relied solely on publicly published data. References of articles used in this scoping review are provided throughout the paper. We have also provided our search terms to enable replication of our search strategy (Table 2).

## References

[nop21271-bib-0001] Abdul Wahab, N. A. , Makmor Bakry, M. , Ahmad, M. , Mohamad Noor, Z. , & Mhd, A. A. (2021). Exploring culture, religiosity and spirituality influence on antihypertensive medication adherence among specialised population: A qualitative ethnographic approach. Patient Prefer Adherence, 15, 2249–2265. Published 2021 Oct 5. 10.2147/PPA.S319469 34675490 PMC8502050

[nop21271-bib-0002] Aghakhani, N. , Sharif, F. , Molazem, Z. , & Habibzadeh, H. (2014). Content analysis and qualitative study of hemodialysis patients, family experience and perceived social support. Iranian Red Crescent Medical Journal, 16(3), e13748. 24829767 10.5812/ircmj.13748 PMC4005429

[nop21271-bib-0003] Akoh, J. A. (2011). Renal transplantation in developing countries. Saudi Journal of Kidney Diseases and Transplantation, 22(4), 637. 21743206

[nop21271-bib-0004] Al‐Ghabeesh, S. , & Suleiman, K. (2014). The lived experience of patients' with end stage renal disease on hemodialysis: A phenomenological study. International Journal of Medicine and Medical Sciences, 47(1), 1423–1429.

[nop21271-bib-0005] Al‐Ghabeesh, S. H. , Alshraifeen, A. A. , Saifain, A. R. , Bashayreh, I. H. , Alnuaimi, K. M. , & Masalha, H. A. (2018). Spirituality in the lives of patients with end stage renal disease; A systematic review. Journal of Religion and Health, 57, 2461–2477. 29671169 10.1007/s10943-018-0622-2

[nop21271-bib-0006] Almutary, H. , Bonner, A. , & Douglas, C. (2016). Which patients with chronic kidney disease have the greatest symptom burden? A comparative study of advanced CKD stage and dialysis modality. Journal of Renal Care, 42(2), 73–82. 10.1111/jorc.12152 26936486

[nop21271-bib-0007] Arksey, H. , & O'Malley, L. (2005). Scoping studies: Towards a methodological framework. International Journal of Social Research Methodology, 8(1), 19–32. 10.1080/1364557032000119616

[nop21271-bib-0008] Australian Bureau of Statistics . (2016). Census data summary. Australian Bureau of Statistics. https://www.abs.gov.au/ausstats/abs@.nsf/Lookup/by%20Subject/2071.0~2016~Main%20Features~Religion%20Data%20Summary~70

[nop21271-bib-0009] Australian Institute Health and Welfare . (2020). Chronic kidney disease. Australian Institute Health and Welfare. Retrieved from https://www.aihw.gov.au/reports/chronic‐kidney‐disease/chronic‐kidney‐disease/contents/how‐many‐australians‐have‐chronic‐kidney

[nop21271-bib-0010] Basińska, M. A. , & Andruszkiewicz, A. (2016). Chronically ill patients' expectations of therapeutic education and their health locus of control. Health Psychology Report, 2(2), 91–102. 10.5114/hpr.2016.55875

[nop21271-bib-0011] Bikbov, B. , Purcell, C. A. , Levey, A. S. , Smith, M. , Abdoli, A. , Abebe, M. , Adebayo O. M. , Afarideh, M. , Agarwal, A. K. , Agudelo‐Botero, M. , Ahmadian, E. , Al‐Aly, Z. , Alipour, V. , Almasi‐Hashani, A. , Al‐Raddadi, R. M. , Alvis‐Guzman, N. , Amini, S. , Andrei, T. , Andrei, C. L. , … Owolabi, M. O. (2020). Global, regional, and national burden of chronic kidney disease, 1990–2017: A systematic analysis for the Global Burden of Disease Study 2017. The Lancet, 395(10225), 709–733. 10.1016/S0140-6736(20)30045-3 PMC7049905 32061315

[nop21271-bib-0012] Biniaz, V. , Moonaghi, H. K. , Froutan, R. , & Ebadi, A. (2018). Subjective adequacy of dialysis; a neglected concept in hemodialysis adequacy. Journal of Renal Injury Prevention, 7(3), 164–170.

[nop21271-bib-0013] Bonner, A. (2007). Understanding the role of knowledge in the practice of expert nephrology nurses in Australia. Nursing & Health Sciences, 9, 161–167. 17688473 10.1111/j.1442-2018.2007.00314.x

[nop21271-bib-0014] Bonner, A. , & Douglas, B. (2018). Principles for nursing practice: Chronic kidney disease. In E. Chang & A. Johnson (Eds.), Living with disability: Principles for nursing practice (3rd ed., pp. 392–406). Elsevier Australia.

[nop21271-bib-0015] Boucquemont, J. , Pai, A. L. , Dharnidharka, V. R. , Hebert, D. , Furth, S. L. , & Foster, B. J. (2019). Gender differences in medication adherence among adolescent and young adult kidney transplant recipients. Transplantation, 103(4), 798–806. 29994983 10.1097/TP.0000000000002359 PMC6326890

[nop21271-bib-0016] Bożek, A. , Nowak, P. F. , & Blukacz, M. (2020). The relationship between spirituality, health‐related behavior, and psychological well‐being. Frontiers in Psychology, 11, 1–13. 32922340 10.3389/fpsyg.2020.01997 PMC7457021

[nop21271-bib-0017] Bragazzi, N. L. , & Puente, G. D. (2013). Chronic kidney disease, spirituality and religiosity: A systematic overview with the list of eligible studies. Health Psychology Research, 1(2), e26. 10.4081/hpr.2013.e26 26973911 PMC4768585

[nop21271-bib-0018] Burke, A. (2008). Could anxiety, hopelessness and health locus of control contribute to the outcome of a kidney transplant? South Africa Journal of Psychology, 38(3), 527–540. 10.1177/008124630803800307

[nop21271-bib-0019] Burker, E. J. , Evon, D. M. , Galanko, J. , & Egan, T. (2005). Health locus of control predicts survival after lung transplant. Journal of Health Psychology, 10(5), 695–704. 10.1177/1359105305055326 16033791

[nop21271-bib-0020] Burlacu, A. , Artene, B. , Nistor, I. , Buju, S. , Jugrin, D. , Mavrichi, I. , & Covic, A. (2019). Religiosity, spirituality and quality of life of dialysis patients: A systematic review. International Urology and Nephrology, 51(5), 839–850. 10.1007/s11255-019-02129-x 30919258

[nop21271-bib-0085] Chen, Y. , & VanderWeele, T. J. (2018, November). Associations of religious upbringing with subsequent health and well‐being from adolescence to young adulthood: An outcome‐wide analysis. American Journal of Epidemiology, 187(11), 2355–2364. 10.1093/aje/kwy142 30215663 PMC6211237

[nop21271-bib-0021] Cousins, M. , Bradshaw, J. , & Bonner, A. (2020). Professional relationships between nephrology clinicians and patients: A systematic review. Journal of Renal Care., 46, 206–215. 32141236 10.1111/jorc.12323

[nop21271-bib-0022] Coyle, J. (2002). Spirituality and health: Towards a framework for exploring the relationship between spirituality and health. Journal of Advanced Nursing, 37(6), 589–597. 11879423 10.1046/j.1365-2648.2002.02133.x

[nop21271-bib-0023] Daugirdas, J. T. (2019). Handbook of chronic kidney disease management (2nd ed.). Wolters Kluwer.

[nop21271-bib-0024] Davison, S. N. , & Jhangri, G. S. (2010). Existential and supportive care needs among patients with chronic kidney disease. Journal of Pain and Symptom Management, 40, 838–843. 20739142 10.1016/j.jpainsymman.2010.03.015

[nop21271-bib-0025] Gill, P. (2012). Stressors and coping mechanisms in live‐related renal transplantation. Journal of Clinical Nursing, 21(11–12), 1622–1631. 22594387 10.1111/j.1365-2702.2012.04085.x

[nop21271-bib-0026] Glover‐Graf, N. M. , Marini, I. , Baker, J. , & Buck, T. (2007). Religious and spiritual beliefs and practices of persons with chronic pain. Rehabilitation Counseling Bulletin, 51, 21–33.

[nop21271-bib-0027] Gültekin, A. , & Kavak Budak, F. (2022). Does spiritual well‐being affect medication adherence in individuals diagnosed with mental illness in Turkey? Journal of Religion and Health, 61, 64–78. 10.1007/s10943-021-01322-6 34213701

[nop21271-bib-0028] Hackett, C. , Cooperman, A. , & Ritchey, K. (2015). The future of world religions: Population growth projections, 2010–2050. Pew Research Center.

[nop21271-bib-0029] Harrington, A. N. N. (2016). The importance of spiritual assessment when caring for older adults. Ageing and Society, 36(1), 1–16. 10.1017/S0144686X14001007

[nop21271-bib-0030] Harris, K. A. , Howell, D. S. , & Spurgeon, D. W. (2018). Faith concepts in psychology: Three 30‐year definitional content analyses. Psychology of Religion and Spirituality, 10(1), 1–29.

[nop21271-bib-0031] Hayes, B. , & Bonner, A. (2010). Job satisfaction, stress and burnout associated with haemodialysis nursing: A review of literature. Journal of Renal Care, 36, 174–179. 20969734 10.1111/j.1755-6686.2010.00194.x

[nop21271-bib-0032] Höllinger, F. , & Makula, L. (2021). Religiosity in the major religious cultures of the world. International Journal of Sociology, 51(5), 345–359.

[nop21271-bib-0033] Hvidt, N. C. , Hvidtjørn, D. , Christensen, K. , Nielsen, J. B. , & Søndergaard, J. (2017). Faith moves mountains—mountains move faith: Two opposite epidemiological forces in research on religion and health. Journal of Religion and Health, 56(1), 294–304. 27541015 10.1007/s10943-016-0300-1 PMC5222926

[nop21271-bib-0034] Kawachi, I. (2020, December). Invited commentary: Religion as a social determinant of health. American Journal of Epidemiology, 189(12), 1461–1463. 10.1093/aje/kwz204 31712820

[nop21271-bib-0035] Johnstone, B. , Franklin, K. L. , Yoon, D. P. , Burris, J. , & Shigaki, C. (2008). Relationships among religiousness, spirituality, and health for individuals with stroke. Journal of Clinical Psychology in Medical Settings, 15(4), 308–313. 10.1007/s10880-008-9128-5 19104988

[nop21271-bib-0036] Jugjali, R. , Yodchai, K. , & Thaniwattananon, P. (2018). Factors influencing spiritual well‐being in patients receiving haemodialysis: A literature review. Renal Society of Australasia Journal, 14, 90–95.

[nop21271-bib-0037] Kidney Health Australia . (2019). Retrieved from http://kidney.org.au

[nop21271-bib-0038] King, P. E. , Kim, S.‐H. , Furrow, J. L. , & Clardy, C. E. (2017). Preliminary exploration of the Measurement of Diverse Adolescent Spirituality (MDAS) among Mexican youth. Applied Developmental Science, 21(4), 235–250.

[nop21271-bib-0039] Kinney, A. Y. , Emery, G. , Dudley, W. N. , & Croyle, R. T. (2002). Screening behaviors among African American women at high risk for breast cancer: Do beliefs about God matter?. In Oncology nursing forum. (Vol. 29, No. 5, pp. 835‐844). Oncology Nursing Society. 12058158 10.1188/02.ONF.835-843

[nop21271-bib-0040] Koenig, H. G. (2012). Religion, spirituality, and health: The research and clinical implications. International Scholarly Research Notices, 2012, 1–34.

[nop21271-bib-0041] Kohli, S. , Batra, P. , & Aggarwal, H. K. (2011). Anxiety, locus of control, and coping stragies among end‐stage renal disease patients undergoing maintenance hemodialsyis. Indian Journal of Nephrology, 21(3), 177–181. 10.4103/0971-4065.83729 21886977 PMC3161435

[nop21271-bib-0042] Kretchy, I. , Owusu‐Daaku, F. , & Danquah, S. (2013). Spiritual and religious beliefs: Do they matter in the medication adherence behaviour of hypertensive patients? BioPsychoSocial Medicine, 7(1), 15. 10.1186/1751-0759-7-15 24138844 PMC3854617

[nop21271-bib-0043] Kripalani, S. , Gatti, M. E. , & Jacobson, T. A. (2010). Association of age, health literacy, and medication management strategies with cardiovascular medication adherence. Patient Education and Counseling, 81(2), 177–181. 20684870 10.1016/j.pec.2010.04.030

[nop21271-bib-0044] Lai, Y. L. , Neo, H. L. M. , Vathsala, A. , & Griva, K. (2020, February). Comparing emotional adjustment of living‐donor and deceased‐donor kidney transplant patients. Transplantation Direct, 6((2), e529. 10.1097/TXD.0000000000000956 32095515 PMC7004627

[nop21271-bib-0045] Lee, Y. M. , Yu, H. Y. , You, M. A. , & Son, Y. J. (2017). Impact of health literacy on medication adherence in older people with chronic diseases. Collegian, 24(1), 11–18. 29218957 10.1016/j.colegn.2015.08.003

[nop21271-bib-0046] Lehmann, A. , Aslani, P. , Ahmed, R. , Celio, J. , Gauchet, A. , Bedouch, P. , Bugnon, O. , Allenet, B. , & Schneider, M. P. (2014). Assessing medication adherence: Options to consider. International Journal of Clinical Pharmacy, 36(1), 55–69. 10.1007/s11096-013-9865-x 24166659

[nop21271-bib-0047] Lin, C.‐C. , Lee, B.‐O. , & Hicks, F. D. (2005). The phenomenology of deciding about hemodialysis among Taiwanese. Western Journal of Nursing Research, 27(7), 915–929. 16275706 10.1177/0193945905278390

[nop21271-bib-0048] Livneh, H. (2016). Quality of life and coping with chronic illness and disability: A temporal perspective. Rehabilitation Counseling Bulletin, 59(2), 67–83. 10.1177/0034355215575180

[nop21271-bib-0049] Mahmoud, S. , & AbdElaziz, N. A. (2015). Associations between health, locus of control, self‐care and self‐efficacy in patients with end stage renal disease unergoing hemodialysis. Life Science Journal, 12(11), 58–78.

[nop21271-bib-0050] Martin, J. C. , & Sachse, D. S. (2002). Spirituality characteristics of women following renal transplantation. Nephrology Nursing Journal, 29, 577–581. 12596606

[nop21271-bib-0051] Maselko, J. R. , Hayward, D. , Hanlon, A. , Buka, A. , & Meador, K. (2012, March 15). Religious service attendance and major depression: A case of reverse causality? American Journal of Epidemiology, 175(6), 576–583. 10.1093/aje/kwr349 22350581 PMC3299417

[nop21271-bib-0087] Mayo‐Gamble, T. L. , & Mouton, C. (2018). Examinging the association between health literacy and medication adherence among older adults. Health Communication, 33(9), 1124–1130. 28636404 10.1080/10410236.2017.1331311

[nop21271-bib-0052] McCann, E. , Donohue, G. , & Timmins, F. (2020). An exploration of the relationship between spirituality, religion and mental health among youth who identify as LGBT+: A systematic literature review. Journal of Religion and Health, 59(2), 828–844 https://doi‐org.acthealthlibrary.idm.oclc.org/10.1007/s10943‐020‐00989‐7 32052279 10.1007/s10943-020-00989-7

[nop21271-bib-0053] Miller, W. R. , & Thoresen, C. E. (2003). Spirituality, religion, and health: An emerging research field. American Psychologist, 58, 24–35. 12674816 10.1037/0003-066x.58.1.24

[nop21271-bib-0054] Moieni, M. , & Eisenberger, N. I. (2018). Effects of inflammation on social processes and implications for health. Annals of the New York Academy of Sciences, 1428(1), 5–13. 29806109 10.1111/nyas.13864 PMC6158086

[nop21271-bib-0055] Moysés Bravin, A. , dos Santos Trettene, A. , de Souza Cavalcante, R. , Burgugi Banin, V. , de Moura Ribeiro Paula, N. A. , Lopes Saranholi, T. , Popim, R. C. , & Modelli de Andrade, L. G. (2017). Influence of spirituality on renal function of kidney transplant patients. Acta Paulista de Enfermagem, 30(5), 504–511. 10.1590/1982-0194201700073

[nop21271-bib-0056] Murgia, C. , Notarnicola, I. , Rocco, G. , & Stievano, A. (2020). Spirituality in nursing: A concept analysis. Nursing Ethics, 27(5), 1327–1343. 32281485 10.1177/0969733020909534

[nop21271-bib-0057] Ohrnberger, J. , Fichera, E. , & Sutton, M. (2017). The relationship between physical and mental health: A mediation analysis. Social Science & Medicine, 195, 42–49. 29132081 10.1016/j.socscimed.2017.11.008

[nop21271-bib-0058] Oji, V. U. , Hung, L. C. , Abbasgholizadeh, R. , Hamilton, F. T. , Essien, E. J. , & Nwulia, E. (2017, April 28). Spiritual care may impact mental health and medication adherence in HIV^+^ populations. HIV/AIDS (Auckland, N.Z.), 9, 101–109. 10.2147/HIV.S126309 PMC5490435 28694708

[nop21271-bib-0059] Olagoke, A. A. , Olagoke, O. O. , & Hughes, A. M. (2021). Intention to vaccinate against the novel 2019 coronavirus disease: The role of health locus of control and religiosity. Journal of Religion and Health, 60, 65–80. 10.1007/s10943-020-01090-9 33125543 PMC7596314

[nop21271-bib-0060] Prihodova, L. , Nagyova, I. , Rosenberger, J. , Majernikova, M. , Roland, R. , Groothoff, J. W. , & Van Dijk, J. P. (2014). Adherence in patients in the first year after kidney transplantation and its impact on graft loss and mortality: A cross‐sectional and prospective study. Journal of Advanced Nursing, 70(12), 2871–2883. 24853863 10.1111/jan.12447

[nop21271-bib-0061] Rafferty, K. A. , Billig, A. K. , & Mosack, K. E. (2015). Spirituality, religion, and health: The role of communication, appraisals, and coping for individuals living with chronic illness. Journal of Religion and Health, 54(5), 1870–1885. 10.1007/s10943-014-9965-5 25341570

[nop21271-bib-0062] Richardson, C. , Percy, M. , & Hughes, J. (2015). Nursing therapeutics: Teaching student nurses care, compassion and empathy. Nurse Education Today, 35, e1–e5. 10.1016/j.nedt.2015.01.016 25682162

[nop21271-bib-0063] Rolley, J. X. , Chang, E. , & Johnson, A. (2018). Spirituality. In E. Chang & A. Johnson (Eds.), Living with disability: Principles for nursing practice (3rd ed., pp.61–72). Elsevier Australia.

[nop21271-bib-0064] Saadatpanah, S. , Zare, N. V. , Maleskzadeh, J. , Sadeghi, T. , & Khorashadizadeh, F. (2018). Relationship between coping and spiritual health in renal transplant recipients. Journal of Evidence‐based Care, 7, 73–77.

[nop21271-bib-0065] Saffari, M. , Lin, C. Y. , Chen, H. , & Pakpour, A. H. (2019). The role of religious coping and social support on medication adherence and quality of life among the elderly with type 2 diabetes. Quality of Life Research, 28(8), 2183–2193. 31037591 10.1007/s11136-019-02183-z

[nop21271-bib-0066] Shahgholian, N. , & Yousefi, H. (2015). Supporting hemodialysis patients: A phenomenological study. Iranian Journal of Nursing and Midwifery Research, 20(5), 626–633. 26457103 10.4103/1735-9066.164514 PMC4598912

[nop21271-bib-0067] Siegel, K. , Anderman, S. J. , & Schrimshaw, E. W. (2001). Religion and coping with health‐related stress. Psychology and Health, 16(6), 631–653.

[nop21271-bib-0068] Siffels, L. E. , Sharon, T. , & Hoffman, A. S. (2021). The participatory turn in health and medicine: The rise of the civic and the need to “give back” in data‐intensive medical research. Humanities and Social Sciences Communications, 8(1), 1–10.

[nop21271-bib-0069] Silva, A. N. , Mortelli, L. , Tavares, P. L. , Marsicano, E. D. O. , Pinhati, R. R. , Colungnati, F. A. B. , Lucchetti, G. , & Sanders‐Pinheiro, H. (2016). Self‐efficacy beliefs, locus of control, religiosity and non‐adherence to immunosuppressive medications in kidney transplant patients. Nephrology (Carlton, Vic.), 21, 938–943. 10.1111/nep.12695 26636921

[nop21271-bib-0070] Sivertsen, N. , Harrington, A. , & Hamiduzzaman, M. (2020). “Two‐eyed seeing”: The integration of spiritual care in Aboriginal residential aged care in South Australia. Journal of Religion, Spirituality & Aging, 32(2), 149–171. 10.1080/15528030.2019.1669515

[nop21271-bib-0071] Tanyi, R. A. (2002). Towards clarification of the meaning of spirituality. Journal of Advanced Nursing, 39(5), 500–509. 10.1046/j.1365-2648.2002.02315.x 12175360

[nop21271-bib-0072] Tarakeshwar, N. , Vanderwerker, L. C. , Paulk, E. , Pearce, M. J. , Kasl, S. V. , & Prigerson, H. G. (2006). Religious coping is associated with the quality of life of patients with advanced cancer. Journal of Palliative Medicine, 9(3), 646–657. 10.1089/jpm.2006.9.646 16752970 PMC2504357

[nop21271-bib-0073] Terpstra, J. , Lehto, R. , & Wyatt, G. (2021). Spirituality, quality of life, and end of life among indigenous peoples: A scoping review. Journal of Transcultural Nursing, 32(2), 161–172. 10.1177/1043659620952524 32851929

[nop21271-bib-0074] Tielen, M. , van Exel, J. , Laging, M. , Beck, D. K. , Khemai, R. , van Gelder, T. , Betjes, M. G. H. , Weimar, W. , & Massey, E. K. (2014). Attitudes to medication after kidney transplantation and their association with medication adherence and graft survival: A 2‐year follow‐up study. Journal of Transplantation, 1–9. 10.1155/2014/675301 PMC4020188 24868449

[nop21271-bib-0075] Tricco, A. C. , Lillie, E. , Zarin, W. , O'Brien, K. K. , Colquhoun, H. , Levac, D. , Moher, D. , Peters, M. D. , Horsley, T. , Weeks, L. , & Hempel, S. (2018). PRISMA extension for scoping reviews (PRISMA‐ScR): Checklist and explanation. Annals of Internal Medicine, 169(7), 467–473. 30178033 10.7326/M18-0850

[nop21271-bib-0076] Tucker, C. M. , Petersen, S. , Herman, K. C. , Fennell, R. S. , Bowling, B. , Pedersen, T. , & Vosmik, J. R. (2001). Self‐regulation predictors of medication adherence among ethnically different pediatric patients with renal transplants. Journal of Pediatric Psychology, 26(8), 455–464. 11700330 10.1093/jpepsy/26.8.455

[nop21271-bib-0077] Umberson, D. , & Karas Montez, J. (2010). Social relationships and health: A flashpoint for health policy. Journal of Health and Social Behavior, 51(1_suppl), S54–S66. 20943583 10.1177/0022146510383501 PMC3150158

[nop21271-bib-0078] Vafaei, A. A. , & Nobahar, M. (2017). The care preferences of patients under hemodialysis. Journal of Renal Injury Prevention, 6(3), 210–215.

[nop21271-bib-0079] Valizadeh Zare, N. , Mohammadi, E. , Zarea, K. , Elahi, N. , & Manzari, Z. (2018). The meaning of coping for kidney transplant recipients: A phenomenological study. Journal of Research in Nursing, 23, 584–595. 34394477 10.1177/1744987118785949 PMC7932060

[nop21271-bib-0080] Vitillo, R. , & Puchalski, C. (2014). World Health Organization authorities promote greater attention and action on palliative care. Journal of Palliative Medicine, 17, 988–989. 25068541 10.1089/jpm.2014.9411

[nop21271-bib-0081] Wanyama, J. , Castelnuovo, B. , Wandera, B. , Mwebaze, P. , Kambugu, A. , Bangsberg, D. R. , & Kamya, M. R. (2007). Belief in divine healing can be a barrier to antiretroviral therapy adherence in Uganda. AIDS (London), 21(11), 1488–1489. 10.1097/QAD.0b013e32823ecf7f 17589198

[nop21271-bib-0082] White, C. , & Gallagher, P. (2010). Effect of patient coping preferences on quality of life following renal transplantation. Journal of Advanced Nursing, 66(11), 2550–2559. 20722812 10.1111/j.1365-2648.2010.05410.x

[nop21271-bib-0083] Yang, F. C. , Chen, H. M. , Pong, S. C. , Chen, C. H. , Wang, S. S. , & Chen, C. M. (2020). Difficulties and coping strategies of kidney‐transplant recipients during their dark postoperative recovery stage after returning home. Transplantation Proceedings, 3226–3230, 2020 Elsevier. 10.1016/j.transproceed.2020.05.011 32636069

[nop21271-bib-0084] Yapa, H. E. , Purtell, L. , Chambers, S. , & Bonner, A. (2020). The relationship between chronic kidney disease, symptoms and health‐related quality of life: A systematic review. Journal of Renal Care, 46(2), 74–84. 10.1111/jorc.12303 31680483

